# Circadian Dysregulation Disrupts Bile Acid Homeostasis

**DOI:** 10.1371/journal.pone.0006843

**Published:** 2009-08-31

**Authors:** Ke Ma, Rui Xiao, Hsiu-Ting Tseng, Lu Shan, Loning Fu, David D. Moore

**Affiliations:** 1 Department of Molecular and Cellular Biology, Baylor College of Medicine, Houston, Texas, United States of America; 2 Department of Pediatrics, Baylor College of Medicine, Houston, Texas, United States of America; Sun Yat-Sen University, China

## Abstract

**Background:**

Bile acids are potentially toxic compounds and their levels of hepatic production, uptake and export are tightly regulated by many inputs, including circadian rhythm. We tested the impact of disrupting the peripheral circadian clock on integral steps of bile acid homeostasis.

**Methodology/Principal Findings:**

Both restricted feeding, which phase shifts peripheral clocks, and genetic ablation in *Per1^−/−^/Per2^−/−^* (PERDKO) mice disrupted normal bile acid control and resulted in hepatic cholestasis. Restricted feeding caused a dramatic, transient elevation in hepatic bile acid levels that was associated with activation of the xenobiotic receptors CAR and PXR and elevated serum aspartate aminotransferase (AST), indicative of liver damage. In the PERDKO mice, serum bile acid levels were elevated and the circadian expression of key bile acid synthesis and transport genes, including Cyp7A1 and NTCP, was lost. This was associated with blunted expression of a primary clock output, the transcription factor DBP, which transactivates the promoters of both genes.

**Conclusions/Significance:**

We conclude that disruption of the circadian clock results in dysregulation of bile acid homeostasis that mimics cholestatic disease.

## Introduction

It has been increasingly recognized that circadian regulation is an integral component of metabolic pathways essential to the maintenance of physiological homeostasis. A variety of metabolic pathways show strong circadian variation that is tightly coupled to both feeding cues and the light-dark cycle [1], [2], [3], [4]. The liver is particularly responsive to circadian rhythm regulation, with many processes under coordinated circadian control, including metabolism of glucose, cholesterol, bile acids and xenobiotics [5]. The key enzymes in both the cholesterol and bile acid metabolic pathways have long been known to show distinct circadian variation in their expression level and enzymatic activity [6], [7], [8], [9], [10], [11]. It has also been well established that bile acid levels are tightly controlled to prevent potential toxicity, ensure appropriate cholesterol catabolism, and promote optimal solubilization and absorption of fat and other essential nutrients. In the liver, bile acid production is a major mechanism for elimination of cholesterol, and the rate limiting enzyme in this process, cholesterol-7α hydroxylase (Cyp7A1), shows distinct circadian expression [10], [11], [12]. The circadian oscillation of the enzyme activity of Cyp7A1 is synchronous with its mRNA level [11]. Furthermore, it was demonstrated that the circadian pattern of Cyp7A1 activity is free-running, persisting under complete darkness, continuous illumination or fasting conditions [10]. Consistent with this, bile flow and biliary secretion of bile acids, cholesterol and phospholipids exhibit distinct daily rhythms [13] that are in accordance with the Cyp7A1 rhythm. Disruption of this coordinately regulated pathway can result in the accumulation of bile acids in the liver, or hepatic cholestasis [14], [15]. A variety of common metabolic abnormalities such as obesity, diabetes and nonalcoholic fatty liver disease are associated with hepatic cholestasis in both humans and animal models [16], [17], [18].

Recently, two nuclear receptor superfamily members that control bile acid homeostasis, farnesoid X receptor (FXR) and small heterodimer partner (SHP), were found to have distinct circadian expression patterns that are linked to their regulation of metabolism [19], [20]. FXR is a bile acid receptor and responds to elevated hepatic bile acid levels by inducing expression of SHP, a potent corepressor of Cyp7A1 expression [21], [22], [23], [24]. Additional bile acid synthetic genes, including Cyp8B1 and Cyp27 are regulated by the FXR - SHP pathway, along with the key transporter NTCP, responsible for uptake of bile acids from the portal circulation. FXR also directly activates expression of BSEP, which mediates export of bile acids into bile [25]. In the intestine, it was found that activation of FXR induces expression of FGF15/19, which in turn represses Cyp7A1 in the liver through FGFR4 [26], [27]. It is clear that coordinated regulation of a number of target genes is necessary to maintain hepatic levels of potentially toxic bile acids within a tight physiological range and disruption of this negative feedback mechanisms leads to cholestasis in the liver as demonstrated by the FXR-null mice [28].

The mammalian circadian system is composed of the hypothalamic suprachiasmatic nucleus (SCN) central clock combined with peripheral clocks in essentially every cell and tissue in the body [5], [20], [29], [30], [31]. In response to light, the SCN central clock initiates and maintains the 24-hour period length throughout the body via synchronization with the clocks in peripheral organs such as the liver [29]. The daily synchronization of central and peripheral clocks is thought to be essential in maintaining normal physiological homeostasis. However, recent studies have revealed that feeding time can act as a dominant Zeitgeber for peripheral tissues. Thus, an experimental strategy of restricted feeding (RF) in which food is provided only during a limited time in the lights-on period in a normal light-dark cycle, when rodents normally do not eat, can dissociate the peripheral clocks from the central clock. RF results in complete phase reversal of hepatic expression of core molecular circadian circuit genes, including Bmal1, Per2, Dbp and Rev-erbα [2], [32], [33]. In mice, RF has been successfully employed to uncouple the central and peripheral clocks to elucidate the effects of metabolic signals on the entrainment of the peripheral clock independent of the central input [32], [33]. Identification of the humoral or metabolic signals involved in the coupling of peripheral clock with the central clock has been an area of intense interest [29], [34], [35]. Various signaling pathways, including glucocorticoid hormones, protein kinase C and calcium are all known to synchronize cells in culture and induce phased circadian gene expression [35]. However, the molecular signal or signals that entrain the peripheral clock in response to RF remain unclear.

Here we address two complementary hypotheses regarding the intersection of circadian rhythm with bile acid homeostasis. In the first, we predicted that since bile acid levels are key downstream targets of circadian control, disruption of such control would result in loss of bile acid homeostasis. Thus, we analyzed the effects of both the dissociation of the central and peripheral clocks by restricted feeding, as outlined above, and also genetic loss of clock function. The genetic approach was based on the period genes (*Per1*, *Per2* and *Per3*), which are integral components of the core molecular circuit that generates the 24 hour rhythm in all the cells of the body [30]. Deletion of either *Per1* or *Per2* results in abnormalities in circadian period length and stability, but both single knockouts maintain circadian rhythms in constant darkness [36], [37], at least for a certain length of time. In contrast, *Per1^−/−^/Per2^−/−^* double knockouts (PERDKO) immediately become arrhythmic upon entry into constant darkness and completely lose daily rhythms of not only locomotor activity but also clock-controlled gene expression [36]. In addition to their role as downstream targets, we hypothesized that bile acids could also be upstream regulators of peripheral clock in the liver. Specifically, since bile acid levels in the enterohepatic circulation fluctuate in response to food intake, which is the zeitgeber in RF, we tested the possibility that FXR dependent bile acid signaling could be an essential component of the food synchronization of peripheral tissues.

We observed that bile acid homeostasis is indeed strongly disrupted by both RF and in the PERDKO model. In contrast, neither loss of FXR nor interruption of enterohepatic circulation of bile acids affected food entrainment. Our results show that intact and synchronized circadian regulation of multiple steps of the entire bile acid metabolic pathway is essential to maintain its homeostasis, and that disruption of such regulation can result in liver stress responses and injury.

## Results

### Bile acid homeostasis is under circadian control

**Figure 1:**
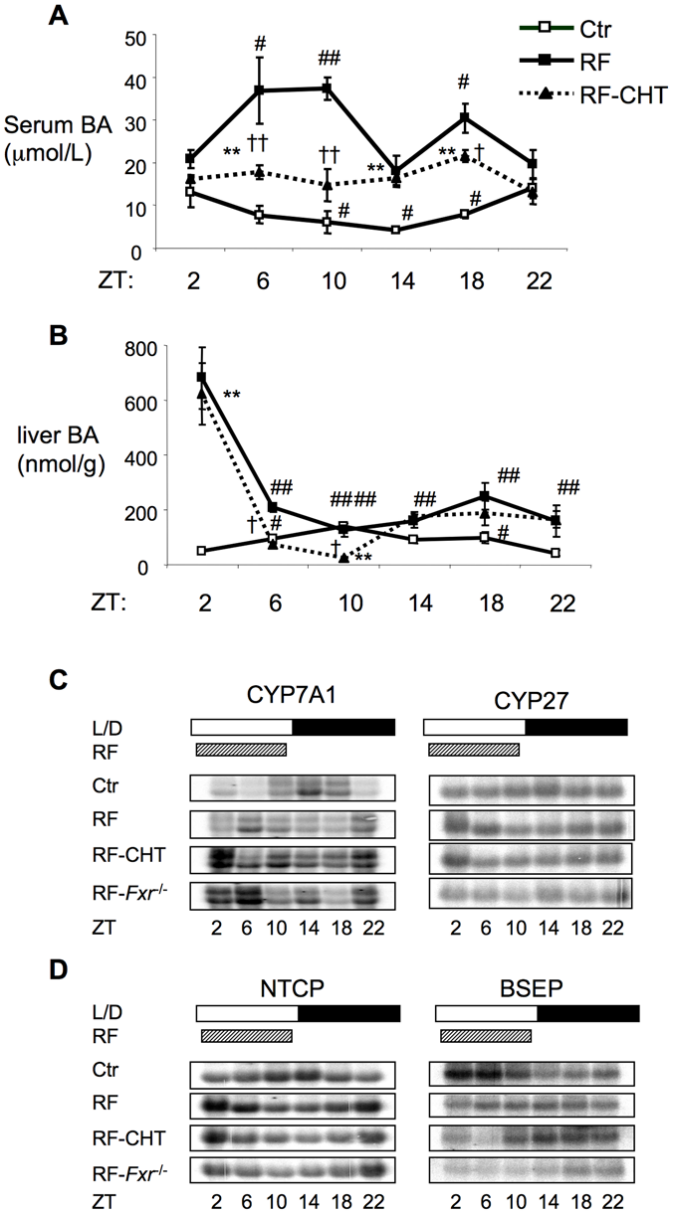
Circadian variation of serum and hepatic bile acid levels and expression of enzymes in mice is reversed by restricted feeding (RF). A. and B. Circadian variations of serum and hepatic bile acid levels in four groups of mice: wild-type mice without restricted feeding (Ctr), wild-type mice under restricted feeding (RF) and wild-type mice on 2% cholestyramine diet under restricted feeding (RF-CHT). Mice were under normal light-dark cycle with lights on at 7∶00 AM (ZT0) and restricted fed from ZT2-ZT10 for 5 days prior to bile acid analysis for the RF groups. Values are expressed as mean ± SEM. n = 6–8 each group for 1A and n = 3–5 for each time points in 1B. * P<0.05 and **P<0.01 compared to Ctr group. † P<0.05 and †† P<0.01 compared to RF group. # P<0.05 and ## P<0.01 compared to RF ZT2 in the same group. C. Circadian expression levels of enzymes involved in bile acid synthesis and D. transporters involved in the uptake and secretion of bile acids in the liver by Northern analysis (n = 3–4 each time point). Loading control 28S is included in Figure SS1C.

We first examined the impact of restricted feeding (RF) on bile acid homeostasis. Serum and hepatic bile acid levels were measured over the 24-hour daily cycle under both normal ad libitum feeding and after 5 days of limited daytime RF (food provided from ZT2 to ZT10). Under normal feeding, serum bile acid levels peak in the early morning hours, decline gradually during the day, and rise again at night. An opposite pattern is observed in the liver, with a peak at approximately ZT10 (Figure 1A, B). Under the RF regimen, serum bile acid levels were significantly elevated, particularly during the daytime period that corresponds to the trough under normal feeding condition. Liver bile acids were also elevated during the night, but showed a dramatic and transient spike in the early morning (ZT2).

To test whether altering specific bile acid signaling pathways in mice would affect the phase shift in serum bile acids and the dysregulation of hepatic bile acid levels we observed in RF, as well as the other responses described below, we used *Fxr* ^−/−^ mice, which have elevated basal bile acid levels due to the loss of negative feedback regulation (Figure S1A and 1B). We also blocked enterohepatic recirculation of bile acids by adding the bile acid binding resin, cholestyramine (CHT), to the normal chow. Loss of FXR function led to even higher serum and hepatic bile acid levels, but did not prevent the reversal of the circadian response during RF (Figure S1A, B). Serum bile acid levels were decreased in the CHT-RF mice compare to RF mice on normal chow, but not to the level of normal control mice, and showed little variation over the 24 hours (Figure 1A, 1B). The diurnal pattern of hepatic bile acid levels in the mice fed the 2% CHT diet was similar to that of the control RF mice, including the ZT2 spike. Thus, our findings demonstrated that even adding 2% CHT to reduce enterohepatic recirculation of bile acids was not able to completely prevent bile acid accumulation in the liver induced by RF.

Next, we examined hepatic expression of four important bile acid metabolic genes under these conditions. Overall, all four genes showed distinct circadian expression patterns that were reversed by RF in the control and also the RF CHT and *Fxr* ^−/−^ mice (Figure 1C, D). Cyp7A1 and Cyp27, two important biosynthetic genes, showed highest expression around 9 PM, at ZT14, under normal conditions. In the RF mice, Cyp7A1 showed 2 apparent peaks in the early morning hours at ZT22 and ZT6, while Cyp27 expression peaked at ZT2. Both loss of FXR and CHT treatment increased the overall level of Cyp7A1 expression, as expected. While there was some variation, the 24 hour expression pattern in both cases was very similar to the control RF mice. Cyp27 expression was not increased in the CHT treated or *Fxr* ^−/−^ mice. The clear phase reversal of the circadian patterns of these genes in response to RF in the control mice was not affected by CHT treatment or the absence of FXR. Similarly, the distinct circadian oscillations of the gene expression of hepatic uptake and export transporters, NTCP and BSEP, were reversed in the control RF mice, and similar responses were also evident in the CHT treated or *Fxr* ^−/−^ mice.

**Figure 2:**
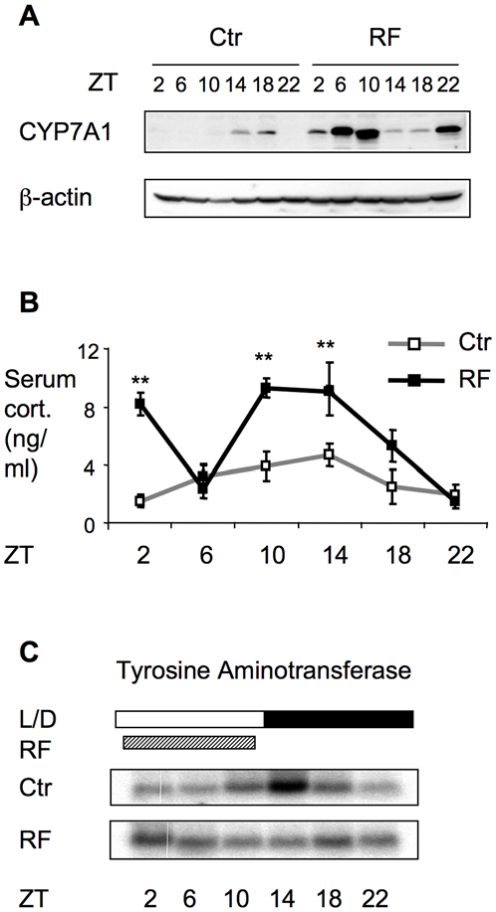
Expression of Cyp7A1 and its correlation with circulating corticosterone level. A. Circadian expression of Cyp7A1 protein level is reversed by restricted feeding as shown by Western analysis. Each time points represents pooled sample of 3–4 mice. B. Circadian variation of serum corticosterone level in control and restricted fed mice. Values are expressed as mean ± SEM and n = 3–4 for each time point. **: p<0.01. C. Expression of tyrosine aminotransferase corresponds to circadian variation of serum corticosterone in Ctr and restricted fed groups as shown by Northern analysis.

The expression of Cyp7A1, the rate-limiting enzyme in bile acid synthesis, was also examined at the protein level of wild-type mice on RF. As expected [11], the overall pattern of Cyp7A1 protein levels generally matched the mRNA levels, but lagged temporally, with peaks at ZT14/18 in the control fed mice, and 2 peaks at ZT6/10 and ZT22 in the RF mice (Figure 2A). Consistent with the increased serum and hepatic bile acid levels in the RF mice, Cyp7A1 protein levels were elevated in the RF mice relative to the control fed mice. Elevated Cyp7A1 protein levels that generally corresponded to mRNA expression were also observed with the CHT-treated and *Fxr* ^−/−^ RF mice (data not shown).

Since Cyp7A1 is transcriptionally regulated by glucocorticoids [12], we investigated whether changes in corticosterone levels could contribute to the RF dependent circadian expression pattern of Cyp7A1. In the normal fed control mice, serum corticosterone peaks at ZT14 of the dark cycle at (Figure 2B), which coincides with the peak of Cyp7A1 mRNA expression. The circadian expression of the glucocorticoid target gene, tyrosine aminotransferase, shows a very similar pattern in the control fed mice (Figure 2C). RF induced a significantly higher corticosterone level that, like Cyp7A1 mRNA and protein expression, showed 2 peaks. This biphasic pattern, which is similar to that previously reported [38], is thought to be due to maintenance of the SCN control of adrenal output in the normal light-dark cycle combined with the additional reverse phase peak due to RF. The first corticosterone peak at ZT2 precedes the initial peak of Cyp7A1 mRNA expression by approximately 4 hours, while the second ZT10/14 peak more closely mirrors the normal fed peak than the peak of Cyp7A1 mRNA expression at 22 hours. Circadian expression of tyrosine aminotransferase was also reversed by RF, with a particularly evident ZT2 tyrosine aminotransferase peak corresponding closely to the early corticosterone peak, but no the later ZT10/14 peak as seen in the normal control. These results suggest that the high corticosterone level of ZT2 in the RF mice contributes to the corresponding peak expression of tyrosine aminotransferase, and likely also the initial peak of Cyp7A1 expression. However, variations in corticosterone levels alone cannot account for the overall circadian expression patterns of either tyrosine aminotransferase or Cyp7A1.

Collectively, these results demonstrate that RF dramatically alters circadian regulation of both bile acid metabolic gene expression and bile acid levels, and that this response is not prevented by CHT treatment or loss of FXR function. RF produces a particularly dramatic spike in hepatic bile acid levels that is preceded by an elevation of Cyp7A1 expression, and is concomitant with increased expression of Cyp27 and the uptake transporter NTCP, as well as decreased expression of the export transporter BSEP.

### Modulating bile acid signaling does not affect hepatic circadian gene expression

**Figure 3:**
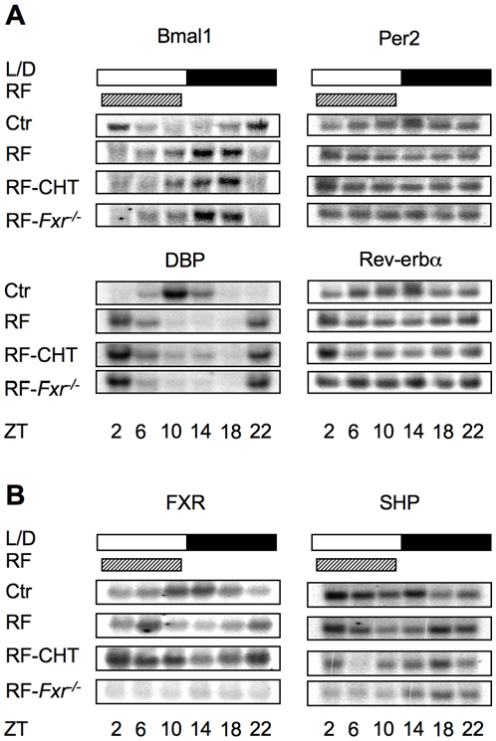
Expression of genes of core circadian rhythm components in liver is not affected by bile acid level in mice. A. Expression of core circadian clock genes in liver is reversed by RF, which is not altered in either the cholestyramine-treated (RF-CHT) or *Fxr* ^−/−^ (RF- *Fxr* ^−/−^) groups. B. Expression of FXR and SHP showed circadian regulation, which is altered by RF.

We examined the response of known components of the circadian clock and its output target genes to RF. As shown in Figure 3A, the expression patterns of Bmal1, Per2, Rev-erbα and DBP were all effectively reversed by RF, as expected [2], [32]. Peak expression of Bmal1 at ZT2/22 was shifted to ZT14/18, while peaks of Per2, DBP and Rev-erbα were switched from ZT10/14 to ZT2/22. The RF dependent reversal was essentially unaffected by either CHT treatment or loss of FXR function, indicating that these changes in bile acid signaling do not alter the entrainment of the hepatic peripheral clock.

We also found that FXR and SHP, two key genes involved in the feedback regulation of Cyp7A1, showed distinct daily rhythms in expression, which were reversed by RF feeding in all the groups examined (Figure 3B). Thus, essentially all of the key components in the regulation of bile acid metabolism, including transcription regulators, major biosynthetic enzymes and transporter proteins, exhibit clear circadian regulation.

Although the tested alterations did not block the RF-induced phase reversal, it remained possible that they could affect the rate of entrainment by RF. Under RF conditions, the phase reversal of the core circadian genes follows a precise time course as exemplified by DBP expression. As previously reported, the peak level of DBP at ZT14 before RF was reversed 12 hours to ZT2 at 3 days of RF, with approximately equal levels between ZT2 and 14 at day one [2], [38]. This phase reversal of DBP expression was not altered by 0.5% cholic acid in the diet or by the absence of FXR function in the *Fxr* ^−/−^ mice, suggesting that bile acid level does not affect the rate of entrainment by RF in the liver (Figure S2). SHP was significantly induced in the CA-fed group, reflecting effectively increased bile acid level but was dramatically decreased in *Fxr* ^−/−^ livers (data not shown). The lack of effect of dietary cholic acid is consistent with a previous study in which long term cholic acid feeding did not affect expression of the primary circadian genes such as Per2, Bmal1 and DBP [39].

### Elevated bile acid results in the induction of liver stress response and injury

**Figure 4:**
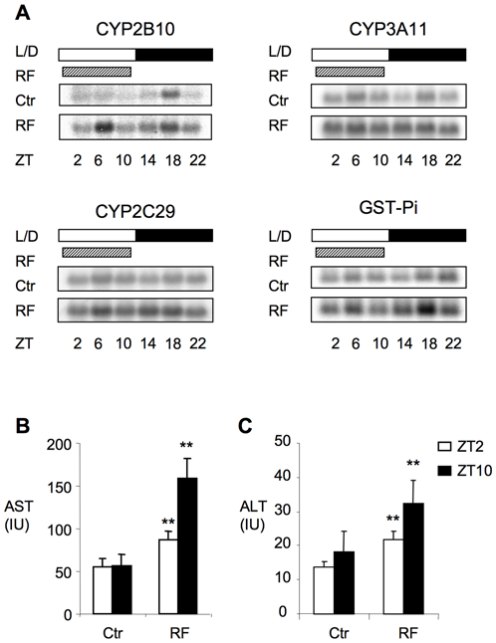
Restricted feeding induction of genes involved in liver stress response and associated liver injury. A. Gene expression and B. serum AST and ALT level (n = 8–10) after 1 week of RF. Values are expressed as mean ± SE. * P<0.05 and **P<0.01 compared to corresponding time point.

High bile acid levels can activate CAR and PXR [40], [41]. Consistent with the markedly increased hepatic bile acid levels at ZT2 (Figure 1A and B), the RF regimen resulted in modest induction of the expression of CAR and PXR target genes, including Cyp2B10, Cyp3A11, Cyp2C29 and GST-Pi (Figure 4A). This was particularly evident for Cyp2B10, a sensitive and responsive CAR target, which showed a transient induction 4 hours after the bile acid peak. Since activation of CAR and PXR signals a stress response of the liver to elevated bile acids, we investigated the physiological consequences of RF. After 1 week of RF (Figure 4B & 4C), both serum AST and ALT were significantly elevated in wild type mice at ZT10, with a lesser but still significant elevation at ZT2, a pattern consistent with transient induction of liver injury due to the elevated bile acids. The moderately elevated basal AST and ALT levels in FXR^−/−^ mice also showed an approximately two-fold further increase in response to 1 week of RF.

### Dysregulation of bile acids in PERDKO

**Figure 5:**
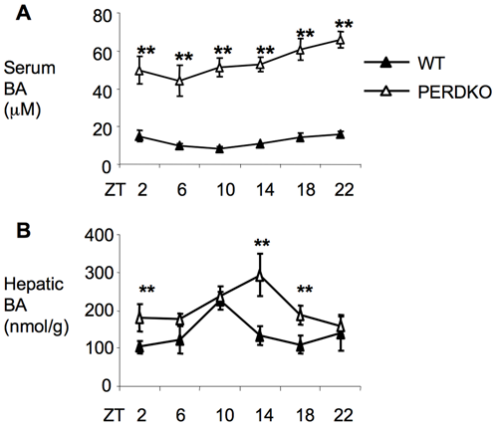
Bile acid dysregulation in PERDKO mice. A. Serum (n = 6–8 each time point) and B. hepatic bile acid levels in PERDKO mice (n = 3–5 each time point). **P<0.01.

We used mice lacking both *Per1* and *Per2* (PERDKO) to ask whether genetic disruption of the circadian clock would affect bile acid homeostasis. Indeed, we found that serum bile acids were markedly elevated in the PERDKO mice, approximately 6–8 fold higher than those of control wild-types throughout ZT2-ZT22 (Figure 5A). In contrast, bile acid levels were not increased in either single knockout (data not shown). Interestingly, hepatic bile acids were also elevated, but were only about 2-fold higher at certain time points, ZT2, 10 and 14 (Figure 5B).

**Figure 6:**
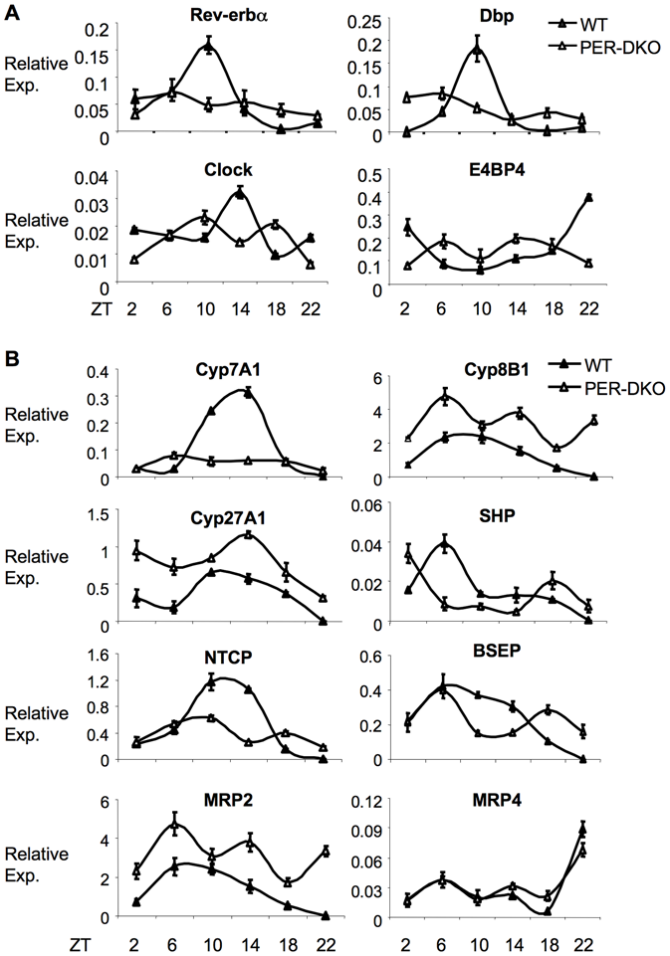
Gene expression changes in PERDKO mice. A. Hepatic expression of genes of the core molecular clock and B. genes involved in bile acid synthesis, transport and regulation in WT and PERDKO measured by quantitative RT-PCR (n = 4 for each time point).

We then analyzed the expression of genes of the molecular clock, bile acid biosynthesis and hepatic transport. Rev-erbα is a component of the negative arm of the circadian transcriptional machinery and has been suggested to be an indirect positive regulator of Cyp7A1 expression by repression of the negative regulators SHP and E4BP4 [42]. In the PERDKO mice, the large amplitude of circadian variation in Rev-erbα expression was lost, with a dramatic decrease in expression at the ZT6 peak. A similar effect was observed with albumin D-element binding protein (DBP), a direct clock controlled output gene that is thought to be the dominant circadian regulator of Cyp7A1 expression [9], [43], [44], [45], with a substantial blunting of the peak expression at ZT10 in the PERDKO mice (Figure 6A). Other core circadian clock genes such as Clock and E4BP4 also showed apparent loss of circadian rhythmic cycling amplitude. Taken together, these data confirm the loss of a functional circadian clock in the liver of the PERDKO mice.

Analysis of the expression of genes involved in bile acid metabolism revealed an explanation for the discrepancy between serum and hepatic bile acid levels in the PERDKO. Consistent with the significantly blunted expression of its positive regulators, DBP and Rev-erbα, daily rhythm of Cyp7A1 expression was completely lost and its overall levels were markedly reduced (Figure 6B). In contrast, Cyp8B1, the enzyme responsible for determining the ratio between the hydrophobic and hydrophilic bile acids composition, exhibited a different pattern in PERDKO. Though the average expression level is higher than that of the wild-type, its rhythmic oscillation pattern was altered. On the other hand, Cyp27A1 and SHP showed a different pattern of expression than the wild-type controls with apparent phase shift and altered expression level.

A major determinant of circulating bile acid concentration lies in the partitioning between portal circulation and hepatic uptake. Since 95% of bile acids secreted into the intestine recycle back to the liver via the portal vein, even small difference in the uptake process could result in significant changes in hepatic and systemic bile acid levels. NTCP is the major transporter in the basolateral membrane of hepatocytes that controls this hepatic uptake process. In the PERDKO livers, NTCP expression was markedly blunted overall, with the loss of its robust circadian peak at ZT10/14. On the apical side of hepatocytes, BSEP is the major transporter responsible for the secretion of bile acids into bile canaliculi. In the double mutant, BSEP circadian pattern is markedly altered, but its expression level on average is not different from that of the wild-type. Other transporters involved in bile acid transport in the liver, such as MRP3 for uptake and MRP2 for excretion were up-regulated, with a loss of circadian cycling, whereas the transporter MRP4 was not altered in the double mutant.

Taken together, our findings of genes involved in bile acid metabolism in the PERDKO mice suggest that overall basolateral extraction of bile acids from portal vein into hepatocytes would be blunted with other transport processes not significantly altered. In addition, bile acid synthesis is also inhibited, while secretion is likely unaffected or decreased. Therefore, the markedly elevated serum with only moderately higher hepatic bile acid levels in the double mutant is primarily due to the decreased hepatic uptake.

### NTCP is a novel DBP target gene

**Figure 7:**
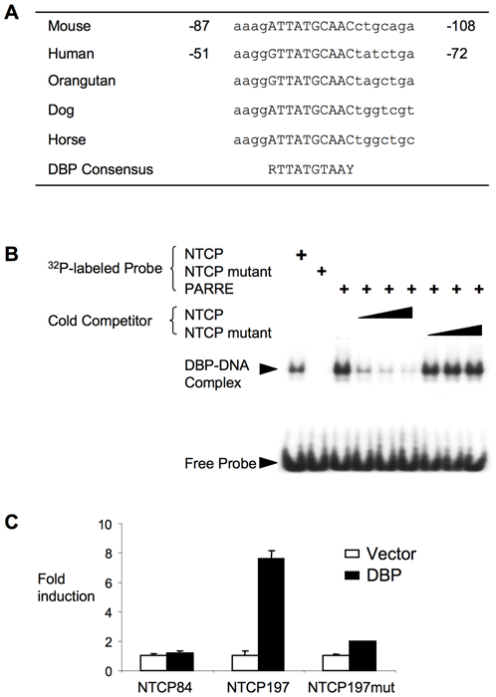
Analysis of circadian regulation of mouse NTCP promoter. A. Sequence alignment of conserved DBP binding site in NTCP promoter region of −87 to −108. R = A or G, Y = C or T in the consensus sequence. B. Gel-shift analysis of DBP binding to DBP putative DBP binding site from the NTCP promoter. Assays were performed with radiolabeled probes containing wild type (NTCP) or mutant (NTCP-mut) DBP binding site. The probe containing high affinity DBP binding consensus sequence (PARRE) was used as positive control. For the competition experiment, cold competitors in excess of 5, 10 and 25 folds were added. Protein-DNA complexes and free probes were indicated by arrowheads. C. Luciferase assay of mouse NTCP promoter constructs with the native and mutated, and without the DBP binding site. NTCP84 contains the region from −84 bp to +37 bp (relative to the transcription starting site), NTCP197 encompass the regions of −197 bp to +37 bp and −1 kb to +37 bp respectively. NTCP197mut was generated by changing 3 nucleotides of the putative DBP binding site (from ATTATGCAAC to ATGGGGCAAC) in the NTCP197 reporter.

Like Cyp7A1, NTCP expression is synchronous with DBP in normal mice, and is decreased in the PERDKO, suggesting that NTCP may also be a direct DBP transcriptional target. Examination of the mouse proximal NTCP promoter region identified a consensus DBP binding site conserved in several mammalian species (Figure 7A). An electrophoretic mobility shift assay confirmed that an oligonucleotide containing the binding site is recognized by DBP, but a point mutant version is not (Figure 7B). Furthermore, a mouse NTCP promoter luciferase reporter construct was strongly transactivated by co-transfection with DBP, and this response was lost in the non-binding point mutant derivative (Figure 7C). We conclude that DBP directly regulates transcription of NTCP, and thereby activates expression of both hepatic bile acid production and uptake.

## Discussion

Hepatic bile acid homeostasis is tightly controlled by a variety of regulators to maintain normal hepatic and serum bile acid concentrations within a relatively narrow range [46], [47]. Our results confirm and extend previous studies by demonstrating that multiple steps of this coordinated physiological process, including bile acid biosynthesis, secretion and uptake, are under precise circadian regulation. We found that disrupting peripheral circadian clocks using either dietary or genetic approaches results in substantial abnormalities of bile acid homeostasis. Uncoupling the peripheral clock from the central clock by RF leads to a substantial, but transient intrahepatic accumulation of bile acids that induces liver stress response and injury, while genetic ablation of both *Per1* and *Per2* results in markedly high serum bile acid levels with moderately elevated hepatic levels.

The nuclear receptors FXR and SHP are key regulators of bile acid homeostasis, coordinately directing the negative feedback regulation of bile acid synthesis through Cyp7A1 [21], [22], [23], [28], and also uptake from circulation through NTCP [48]. The FXR-SHP cascade functions in concert with the FXR dependent feed forward induction of secretion into the bile through BSEP [49], which is thought the be a primary determinant of bile flow [50]. Disruption of bile acid homeostatic regulation by deletion of FXR leads to elevation in hepatic bile acid levels that results in activation of CAR and PXR and damage to hepatocytes [28], [51]. Our results show that a more dramatic, but transient bile acid accumulation as a result of dys-synchronization of the peripheral and central clocks not only activates CAR and PXR target genes, but also increases serum liver enzyme levels, demonstrating a moderate level of liver damage. Initial results indicate that this response declines as the liver adapts to the RF regimen (Figure S3). After 2 weeks of continuous RF, the elevation of serum and liver BA levels at ZT2 and ZT10 is approximately half of that observed at 1 week, and AST levels are only modestly increased. At 4 weeks, a modest increase in hepatic bile acid levels is still present at both ZT2 and ZT10, but serum bile acid and AST levels are trended down to within normal range.

The ZT2 peak in hepatic bile acid induced by RF is due to the convergence of several factors. Cyp7A1 protein levels are elevated overall, and the ZT2 peak is preceded by an increase in Cyp7A1 gene expression and protein levels. It also coincides with both a decrease in the expression of the efflux transporter BSEP, and an increase in the uptake transporter NTCP. Collectively, all three of these effects could contribute to increased bile acid levels within the hepatocyte. The molecular mechanisms that account for these responses are less clear, due to the complexity of the regulatory inputs, the feedback cycles involved, and the dynamic nature of the response. Cyp7A1 is a known DBP target gene [9], whereas our current results identify NTCP as a novel DBP target. In addition, glucocorticoids was shown to be able to induce expression of both Cyp7A1 [52] and NTCP [53]. Thus, it is likely that increases in both DBP expression and glucocorticoid levels contribute to the increased levels of Cyp7A1 and NTCP around the ZT2 time point.

The PERDKO mice showed loss of peripheral clock in the liver in animals maintained in a normal 24 hour light dark cycle, as evidenced by expression patterns of multiple core and output clock genes. This was particularly evident for both DBP and Rev-erbα, which showed markedly blunted overall expression in addition to loss of rhythmicity. The deficit in DBP expression contributes to the decreased expression of both Cyp7A1 and NTCP, and Rev-erbα has recently been shown to activate at least the former [42]. Defects in circadian regulation of Cyp7A1 in *Clock* ^−/−^ mice were also linked to dramatically decreased expression of DBP and Rev-erbα [39], [54]. In turn, the significantly reduced NTCP expression provides a direct explanation for the markedly elevated circulating bile acid in the PERDKO mice and the discordance between hepatic and serum levels.

Taken together, our results establish that bile acid biosynthesis, transport and secretion are all downstream targets of circadian regulation in the liver. Specific inputs from glucocorticoids and DBP coordinate an increase in liver bile acids by activating expression of both Cyp7A1 and NTCP in the RF model. While the circadian response of Cyp7A1 is known, we are not aware of any descriptions of similar regulation for NTCP, which we have identified as a novel direct DBP target. Partitioning of bile acids in portal circulation and systemic circulation is dependent on the percentage of hepatic uptake [55] mediated by NTCP. Thus, its dysregulation can substantially affect hepatic and systemic bile acid levels, as observed in the PERDKO mice. The observation that bile flow shows a distinct circadian pattern [13] is also consistent with our result demonstrating a substantial circadian pattern of BSEP expression. It is apparent that the complex pathways of bile acid homeostasis are dependent on proper circadian control, and that loss of such control can lead to deleterious effects, including liver injury.

Our results did not support a dominant role for bile acids as upstream regulators of the peripheral clock. Specifically, the efficient entrainment of the hepatic clock by RF was not affected by loss of FXR function, interruption of enterohepatic circulation, or increasing the bile acid pool. The failure of streptozotocin induced diabetes to prevent RF entrainment indicates that food induced insulin responses are also not essential for this effect [56]. In contrast, exogenous glucocorticoid treatments can directly phase shift the peripheral clock in mice [34], and altering glucocorticoid signaling alters the rate of RF entrainment [38]. Diverse signals including serum shock, cAMP, protein kinase C, glucocorticoids and Ca^2+^ can induce a transient surge of Per1 expression and phased circadian gene expression in cultured cells [35]. Thus, multiple reinforcing signals, rather than a single dominant factor, may drive entrainment in response to RF.

We conclude that coordinated circadian regulation of individual steps of bile acid metabolism is essential to achieve the tight physiological homeostasis required to prevent the development of cholestatic liver disease, which adds yet another layer of control over the intricacy of regulation of bile acid metabolism. As demonstrated by our RF regimen in mice, the dysynchronization of the central and peripheral clock created by timing of the meals has deleterious effects in the liver.

There are interesting parallels between the rodent RF regimen and the circadian disruption experienced by shift workers, who have variable eating and activity schedules in the context of a constant 24 hour light-dark cycle. For example, night-shift workers consistently eat more during the night even on their off-shift days [57]. In a study of a large cohort of Japanese railway workers, those who were “involved in any night shift working” had an odds ratio of 4.7 (95% confidence intervals 2.7, 8.1) for AST levels greater than 2-fold above the upper limit of normal [58]. A smaller study also associated shift work with elevated ALT levels [59]. Our results raise the intriguing possibility that disruption of bile acid homeostasis and the resultant accumulation of bile acids in the liver might contribute to complaints of gastrointestinal distress that have long been among the best known health complications of shift work [60], [61]. It is conceivable that synchronizing mealtimes with light-dark and sleep-activity cycles could minimize detrimental health effects of night-shift work or jet lag.

## Materials and Methods

### Animals

Animals were maintained in the Baylor College of Medicine Transgenic Mice Facility under a constant 12∶12 light dark cycle, with light on at 7∶00AM (ZT0). All experiments were done following approval of the protocol by the animal care research committee of Baylor College of Medicine. *Fxr^−/−^* and *SHP^−/−^* mice were generated and maintained as described [23], [28]. *Per1^−/−^/Per2^−/−^* double knockout mice (PERDKO) were generated by cross breeding, as described [36], [37]. For the restricted feeding regimen, mice were placed in a normal L/D cycle with food taken out during lights out (ZT10-ZT2) and free access to food during lights on (ZT2-ZT10).

### Serum Analysis

Blood was collected by retro-orbital bleed at the indicated time points and plasma was isolated and kept frozen at −20°C until analysis. Plasma bile acid levels were determined by bile acid assay kit (Diagnostic Chemicals Ltd.). Glucose, cholesterol and triglyceride kits were obtained from Thermo DMA Inc. ALT kits were obtained from TECO diagnostics. Corticosterone level was determined using EIA method (Cayman Chemical).

### Hepatic Bile Acid Analysis

Bile acids were extracted from frozen liver tissue by homogenization in 75% ethanol and incubation at 50°C for 2 hrs as described [62]. The extracted supernatant was assayed using the same bile acids kit as above after dilution of 1∶5 with PBS. The concentration was normalized to grams of liver tissue used.

### RNA Extraction, Quantitative Reverse-Transcriptase PCR and Northern analysis

Total RNA was isolated from snap-frozen liver tissues using Trizol reagent (Invitrogen Corp.). cDNA was generated using Omniscript kit (Qiagen) and quantitative PCR was performed and analyzed using an Applied Biosystems 7700 with FastStart SYBR green master mix (Roche Diagnostics). Relative expression levels were determined using the comparative Ct method to normalize target gene to actin. Northern blot analysis were performed as previously described using 20 ug of total RNA fractionated on 1.2% 2 M formaldehyde agarose gels and transferred to Zeta-Probe GT genomic membrane (Bio-Rad). Gene-specific cDNA probes amplified by RT-PCR were used for hybridization in UltraHyb buffer (Ambion) at 42°C overnight.

### Western Blot Analysis

80 µg of total protein from liver tissue homogenates were used for each sample on SDS-PAGE gel. Protein were then transferred to nitrocellulose membrane after electrophoresis, blotted using specific primary and secondary antibody and detected by chemiluminescence (Supersignal; Pierce Biotechnology). Rabbit polyclonal Cyp7A1 (H-58) antibody and actin antibody were obtained from Santa Cruz Biotechnology, Inc.

### NTCP Promoter Analysis

Full-length mouse DBP cDNA was cloned into pCMX vector. Mouse NTCP promoter regions were amplified from mouse genomic DNA and cloned into pGL3 vector (Promega). NTCP84 and NTCP197 contains the region from −84 bp to +37 bp (relative to the transcription starting site) and −197 bp to +37 bp respectively. NTCP197mut was generated by changing 3 nucleotides of the putative DBP binding site (from ATTATGCAAC to AT**GGG**GCAAC) in the NTCP197 reporter. HepG2 cells were transfected with Fugene HD (Roche) following manufacturer's instruction. Each well was transfected with 400 ng pCMX-DBP or pCMX empty vector, 100 ng pGL3 reporter plasmids and 50 ng β-galatosidase for normalization. Forty eight hours after transfection, cells were lysed in Tropix lysis buffer and lysate was assayed for luciferase activities.

### Gel-shift Assay

Gel shift assay: Double-stranded oligonucleotide probes were generated by annealing two oligonucleotides and filling 5′-overhang with Klenow exo^−^ large fragment in the presence of [α-^32^P]dCTP. Competitors were generated with the same method by replacing the radioactive dCTP with cold dCTP. Oligonucleotide sequences were:

NTCP-fwd: 5′-CAAATAAAAGATTATGCAAC

NTCP-rev: 5′-GTTCTGCAGGTTGCATAATCTTTTATTTG

NTCPmut-fwd: 5′-CAAATAAAAGAT**ggg**GCAAC

NTCPmut-rev: 5′-GTTCTGCAGGTTGC**ccc**ATCTTTTATTTG

PARRE-fwd: 5′-AACCCATTGGAGATTACGTAA

PARRE-rev: 5′-GTTCTTGGTTACGTAATCTCCAATGGGTT

The NTCP probe contains the DBP binding site and flanking sequence from the mouse NTCP promoter region and three nucleotides were mutated in the NTCPmut probe. The PARRE probe contains the high affinity DBP binding site (Lopez-Molina et al., EMBO, 1997). Mouse DBP was in vitro translated using the TNT Quick Coupled Transcription/Translation system (Promega) following manufacturer's instruction. Binding reactions were performed by combining 2 µl in vitro translated DBP and 100 fmole ^32^P-labeled probe with or without cold competitors in a 20 µl volume of 20 mM HEPES (pH7.9), 50 mM KCl, 5 mM MgCl_2_, 0.5 mM EDTA, 10% glycerol, 0.5 mM DTT, 1 mM PMSF and 50 ng/µl poly(dI-dC). After 20 minutes incubation at room temperature, protein-DNA complexes were resolved on a 5% polyacrylamide gel in 0.25xTBE buffer.

### Statistical Analysis

All results are expressed as mean±SEM. Data were analyzed by unpaired two-tailed Student's t test. P<0.05 was considered statistically significant.

## Supporting Information

Figure S1 (0.83 MB TIF)

Figure S2 (0.83 MB TIF)

Figure S3 (0.67 MB TIF)
