# Supplementary figures and images for: Circadian Dysregulation Disrupts Bile Acid Homeostasis
Source: PLoS One. 2009 Aug 31;4(8):e6843. doi: 10.1371/journal.pone.0006843 (PMC2730029; doi:10.1371/journal.pone.0006843)

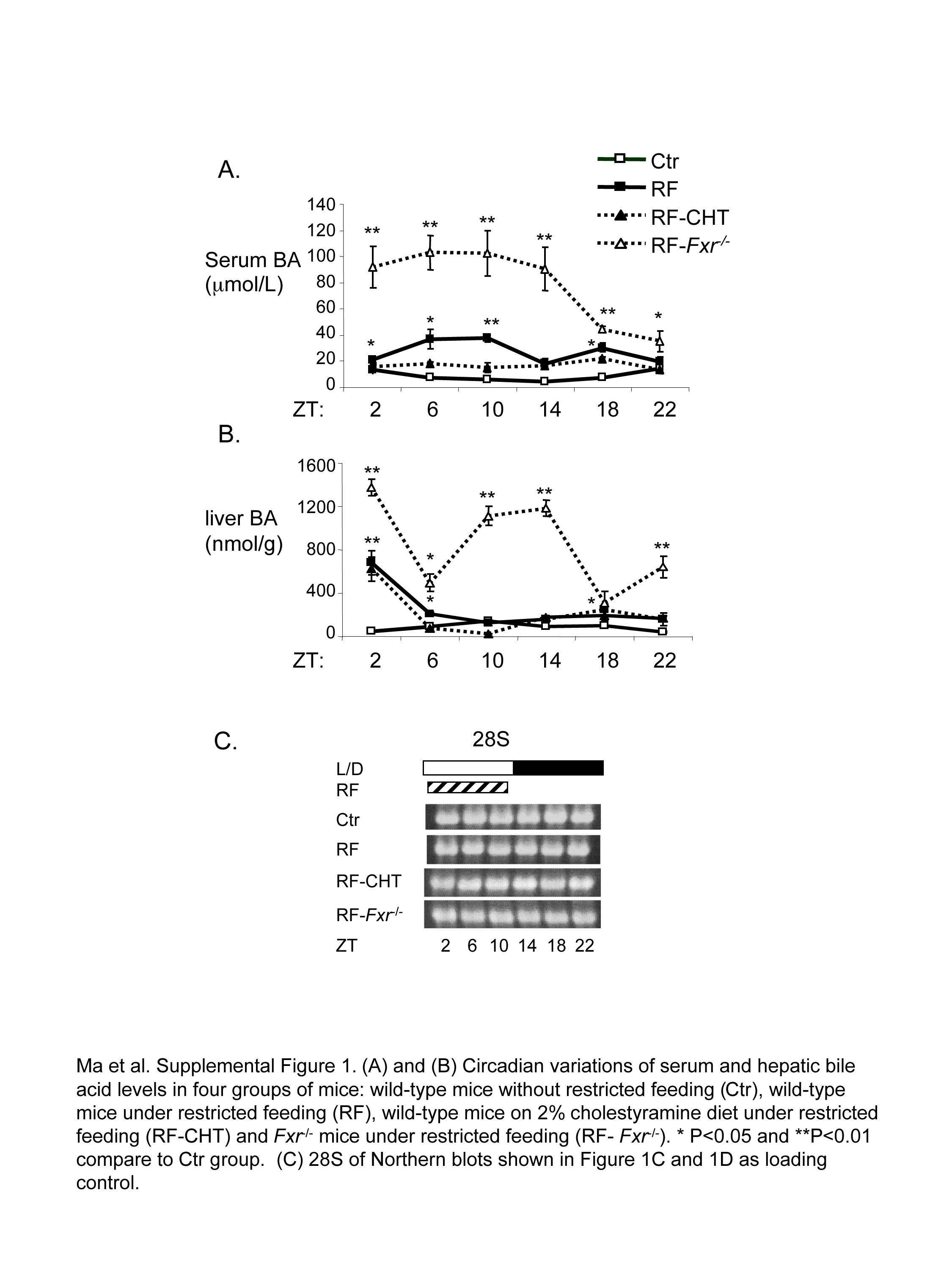

Supplement: Figure S1 — (0.83 MB TIF) [file pone.0006843.s001.tif]

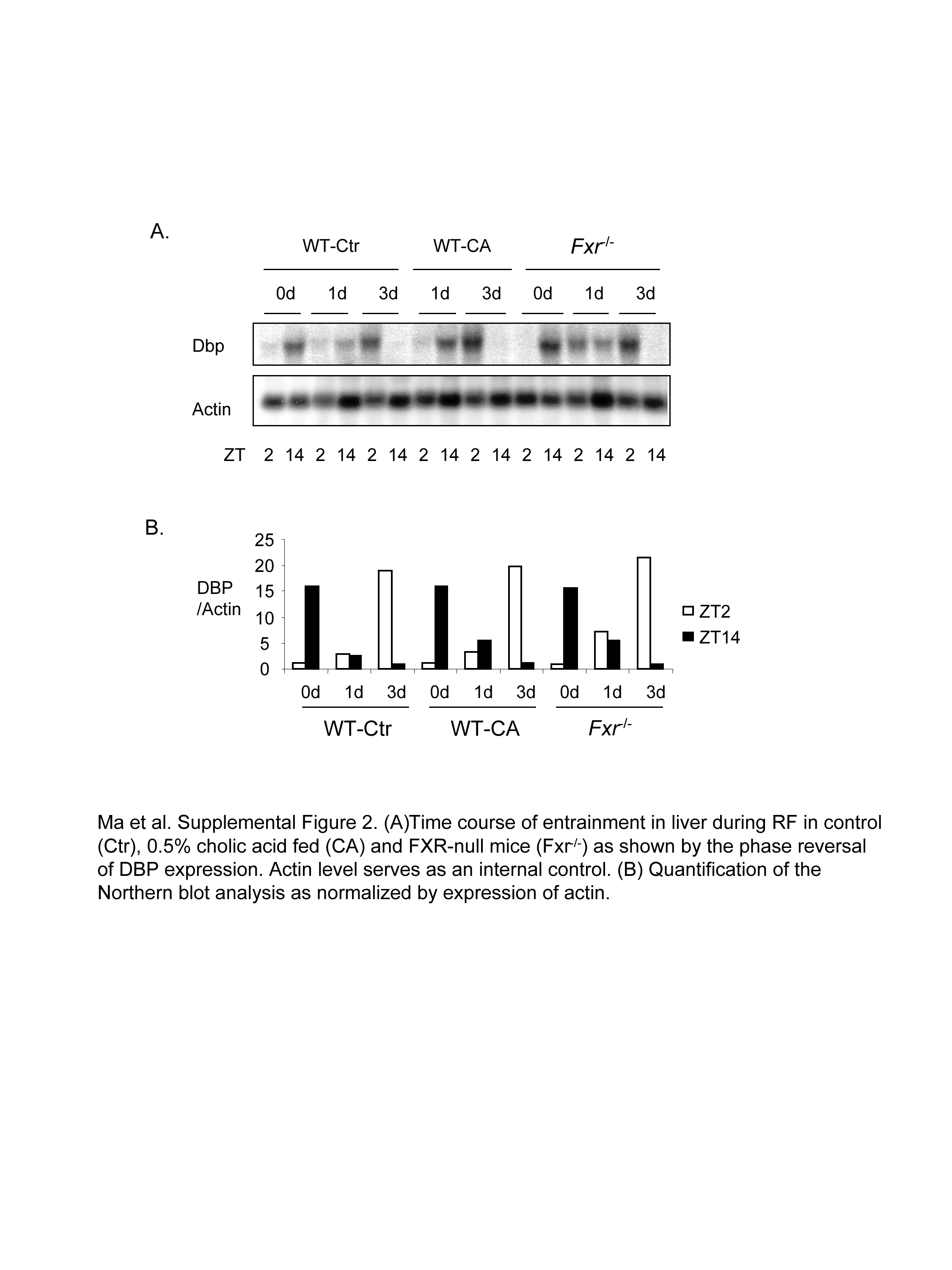

Supplement: Figure S2 — (0.83 MB TIF) [file pone.0006843.s002.tif]

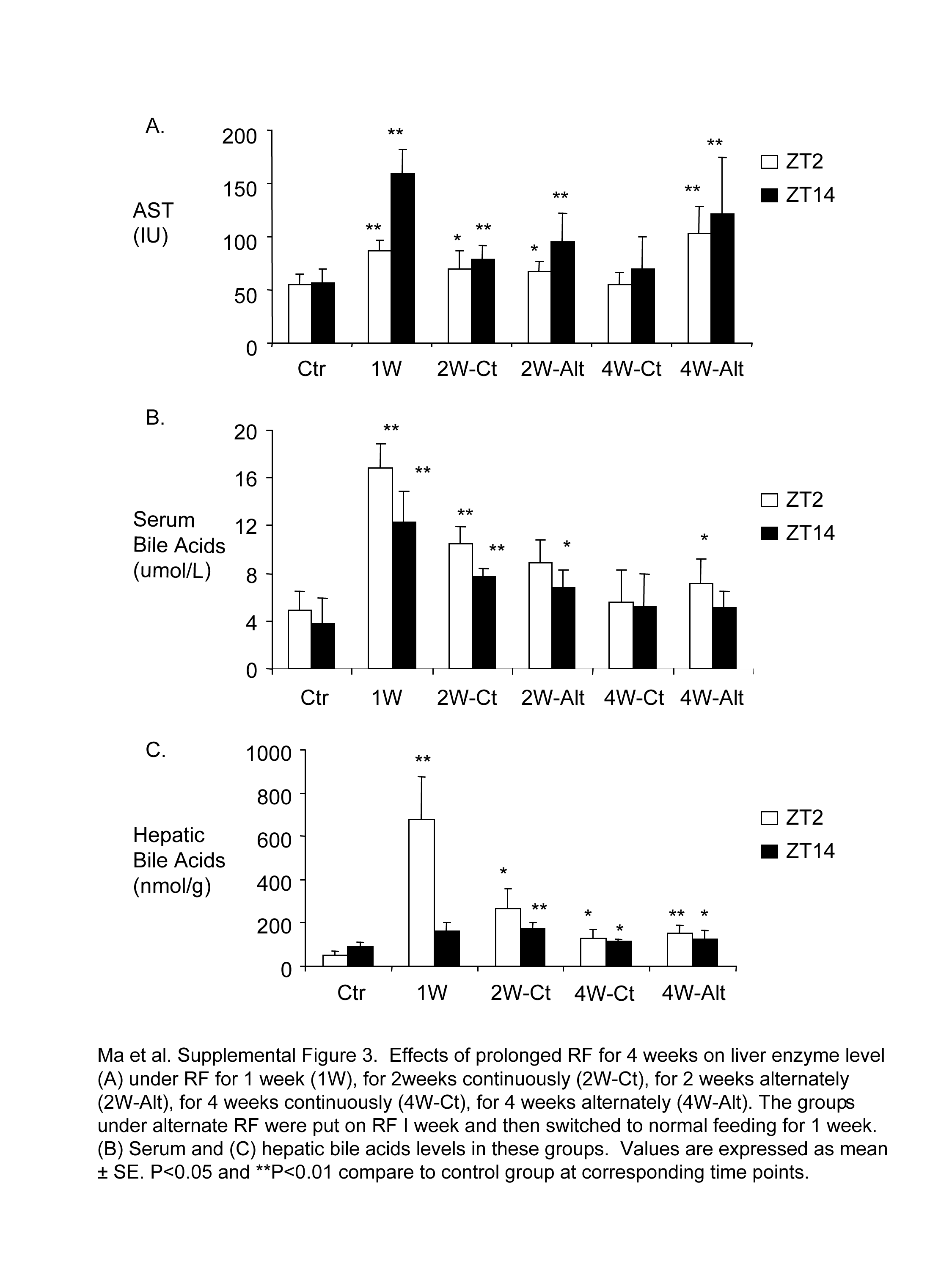

Supplement: Figure S3 — (0.67 MB TIF) [file pone.0006843.s003.tif]
